# Controlled Activity of the *Salmonella* Invasion-Associated Injectisome Reveals Its Intracellular Role in the Cytosolic Population

**DOI:** 10.1128/mBio.01931-17

**Published:** 2017-12-05

**Authors:** Jessica A. Klein, Jesse R. Grenz, James M. Slauch, Leigh A. Knodler

**Affiliations:** a Paul G. Allen School for Global Animal Health, College of Veterinary Medicine, Washington State University, Pullman, Washington, USA; b Department of Microbiology, University of Illinois at Urbana-Champaign, Urbana, Illinois, USA; University of British Columbia

**Keywords:** *Salmonella enterica*, intracellular proliferation, type III secretion, vacuoles

## Abstract

The *Salmonella* invasion-associated type III secretion system (T3SS1) is an essential virulence factor required for entry into nonphagocytic cells and consequent uptake into a *Salmonella*-containing vacuole (SCV). While *Salmonella* is typically regarded as a vacuolar pathogen, a subset of bacteria escape from the SCV in epithelial cells and eventually hyperreplicate in the cytosol. T3SS1 is downregulated following bacterial entry into mammalian cells, but cytosolic *Salmonella* cells are T3SS1 induced, suggesting prolonged or resurgent activity of T3SS1 in this population. In order to investigate the postinternalization contributions of T3SS1 to the *Salmonella* infectious cycle in epithelial cells, we bypassed its requirement for bacterial entry by tagging the T3SS1-energizing ATPase InvC at the C terminus with peptides that are recognized by bacterial tail-specific proteases. This caused a dramatic increase in InvC turnover which rendered even assembled injectisomes inactive. Bacterial strains conditionally expressing these unstable InvC variants were proficient for invasion but underwent rapid and sustained intracellular inactivation of T3SS1 activity when InvC expression ceased. This allowed us to directly implicate T3SS1 activity in cytosolic colonization and bacterial egress. We subsequently identified two T3SS1-delivered effectors, SopB and SipA, that are required for efficient colonization of the epithelial cell cytosol. Overall, our findings support a multifaceted, postinvasion role for T3SS1 and its effectors in defining the cytosolic population of intracellular *Salmonella*.

## IMPORTANCE

A needle-like apparatus, the type III secretion system (T3SS) injectisome, is absolutely required for *Salmonella enterica* to enter epithelial cells; this requirement has hampered the analysis of its postentry contributions. To identify T3SS1-dependent intracellular activities, in this study we overcame this limitation by developing a conditional inactivation in the T3SS whereby T3SS activity is chemically induced during culture in liquid broth, permitting bacterial entry into epithelial cells, but is quickly and perpetually inactivated in the absence of inducer. In this sense, the mutant acts like wild-type bacteria when extracellular and as a T3SS mutant once it enters a host cell. This “conditional” mutant allowed us to directly link activity of this T3SS with nascent vacuole lysis, cytosolic proliferation, and cellular egress, demonstrating that the invasion-associated T3SS also contributes to essential intracellular stages of the *S. enterica* infectious cycle.

## INTRODUCTION

Intracellular bacteria face unique challenges in overcoming innate host defenses, irrespective of the intracellular niche that they occupy. For intracellular pathogens that inhabit a membrane-bound vacuole (e.g., *Yersinia*, *Chlamydia*, *Mycobacterium*, *Salmonella*, and *Brucella* species), survival depends on their ability to modulate trafficking of the phagosome to prevent acidification and/or fusion with lysosomes, whereas pathogens that lyse their internalization vacuole and proliferate in the cytosol (e.g., *Shigella*, *Listeria*, *Francisella*, and *Rickettsia* species) must be able to avoid or defend against cytosolic host defense mechanisms, such as autophagy and inflammasomes. The ability of bacteria to direct themselves to a specific intracellular locale is key to their pathogenesis, as this ability not only determines their survival and proliferation but also, ultimately, their virulence. For Gram-positive pathogens, escape from the internalization vacuole has been relatively well-characterized and is often dependent on pore-forming toxins that destabilize the vacuolar membrane, thereby permitting their escape (1–4). In general, Gram-negative bacteria do not encode such toxins, and molecular determinants for how they remain confined within a phagosome or rupture this compartment remain ambiguous.

Also known as the injectisome, type III secretion systems (T3SS) are widespread in Gram-negative pathogens, and they are composed of over 20 different proteins (5). They act as a molecular syringe to deliver type III effectors directly from the bacterial cytosol into the host cell cytosol, thereby modulating a variety of host cell functions. A *Shigella flexneri* type III effector, IpgD, has previously been implicated in the efficiency of bacteria-containing vacuole lysis (6). T3SS have inherent pore-forming activity via insertion of the translocon, a protein complex composed of two translocator proteins, into eukaryotic membranes (7). The translocator proteins themselves also influence the magnitude of bacteria-containing vacuole lysis, in a yet-to-be-defined manner (8). Finally, coat protein complex II (COPII) function, which is required for protein export from the endoplasmic reticulum, also influences the efficiency of nascent *Salmonella*-containing vacuole lysis, albeit by an unknown mechanism (9). Sec13, one of the five core components of the COPII complex, does not appear to be involved in rupture of the *Shigella*-containing vacuole, however (6). Collectively, these studies allude to a complex interplay of bacterial and host cell proteins that determine the intracellular localization of a particular Gram-negative pathogen.

The gastrointestinal pathogen *Salmonella enterica* serovar Typhimurium, occupies an intracellular niche in diverse host cell types, including epithelial cells and macrophages. Its pathogenic traits are largely dictated by two T3SS, T3SS1 and T3SS2, which are encoded on *Salmonella* pathogenicity island 1 (SPI1) and SPI2, respectively, and are spatiotemporally regulated. T3SS1 is necessary for bacterial entry into nonphagocytic cells (10); it delivers several type III effectors that drive localized plasma membrane ruffling, which allows the membrane to envelop and internalize the bacterium (11); this is known as a “trigger” mechanism of entry (12). The genes encoding T3SS1 are induced extracellularly and downregulated after internalization (13, 14). Genes encoding T3SS2 are induced intracellularly (15), in agreement with its requirement for modulation of *Salmonella*-containing vacuole (SCV) trafficking, SCV membrane integrity, and bacterial intracellular survival and replication (15–18). Although classically defined as a vacuolar pathogen, *S*. Typhimurium also lyses its internalization vacuole (19, 20), albeit much less efficiently than “typical” cytosolic pathogens and by a mechanism that remains unclear. The efficiency of nascent vacuole lysis varies considerably and is generally higher in epithelial cells than in macrophages: 10 to 20% and 2 to 6% of *S*. Typhimurium cells lyse their internalization vacuole within 90 min postinfection (p.i.) in epithelial cells and macrophages, respectively (8, 20, 21, 22) (see Fig. S1 in the supplemental material). The fate of *S*. Typhimimurium in the cytosol is also host cell type dependent. In the cytosol of macrophages and fibroblasts, growth of *Salmonella* is inhibited by host cysteine proteases, specifically, caspase-1 and caspase-11 (16, 22). In contrast, *Salmonella* replicates to high numbers in the cytosol of epithelial cells, eventually filling the entire cytosolic space and causing cell death by pyroptosis (19). These hyperreplicating bacteria are flagellated, motile, and induced for T3SS1 genes (19, 20, 23), suggesting prolonged or resurgent activity of T3SS1 in this cytosolic population. Although expressed, it is unclear whether T3SS1 is required for colonization of the host cell cytosol.

10.1128/mBio.01931-17.1 FIG S1  Effect of InvC destabilization in cell lines with differential requirements for T3SS1-mediated entry. (A) Invasion efficiency of a T3SS1 mutant (Δ*prgI*), determined in a gentamicin protection assay in the indicated cell lines at 1 h p.i. and expressed as the percentage of the invasion efficiency for wild-type bacteria in each cell line (which was set to 100%). HCT116, human colonic epithelial cells; HeLa, human cervical epithelial cells; C2BBe1, human colonic epithelial cells; J774A.1, mouse macrophage-like cells. (B) CHQ resistance assay results in various cell lines. Bacterial subcultures were grown in the presence of 100 ng/ml ATc for 1.5 h prior to infection to induce synthesis of InvC-FLAG. MOIs were adjusted so that equivalent numbers of bacteria were internalized, and the proportion of cytosolic bacteria was determined at the indicated time points in a CHQ resistance assay. Data are means ± SD (*n* ≥3 independent experiments). Asterisks denote results significantly different from those for wild-type bacteria. Download FIG S1, PDF file, 1.1 MB. Copyright © 2017 Klein et al. 2017 Klein et al. This content is distributed under the terms of the Creative Commons Attribution 4.0 International license.

The ability to assess postinvasion roles of T3SS1 in nonphagocytic cells has been hampered by its requirement for bacterial internalization, i.e., T3SS1 mutants are invasion defective. Previously, two methods have typically been used to bypass this T3SS1-mediated entry requirement. The first involves rerouting internalization through T3SS1-independent mechanisms, either by using nonphagocytic cell lines that are relatively permissive for T3SS1-independent entry (8, 20) or via surface expression of invasin from *Yersinia pseudotuberculosis* (21, 24, 25), which forces an *S*. Typhimurium T3SS1 mutant to utilize a “zipper” mechanism of entry (12). The second method relies on cointernalization of T3SS1 mutants with wild-type *S*. Typhimurium (25). Collectively, these studies have suggested a role for T3SS1 in both biogenesis and lysis of the nascent SCV (20, 21, 25). Given our recent finding that the T3SS translocator proteins affect early trafficking events of bacteria-containing vacuoles (8), here we wished to examine the postinvasion contributions of T3SS1 while maintaining the native T3SS1-dependent trigger mode of bacterial internalization and biogenesis of the nascent SCV. We targeted a number of T3SS1-associated genes and found that one was particularly amenable to absolute and continual intracellular inactivation: the T3SS1-energizing ATPase InvC. When InvC was genetically engineered to have a C-terminal peptide tag that dramatically shortened its half-life and when its expression was placed under the control of an anhydrotetracycline (ATc)-inducible promoter, T3SS1 activity was robust in the presence of ATc but lacking in the absence of inducer. This allowed us to directly pinpoint new intracellular processes that are dependent on T3SS1 activity, including cytosolic proliferation and subsequent bacterial egress. We further identified two T3SS1 effectors, SopB and SipA, which contribute to bacterial proliferation in the epithelial cell cytosol. Collectively, this work demonstrates that the invasion-associated T3SS also contributes to essential intracellular stages of the *S. enterica* infectious cycle.

## RESULTS

### Establishing a system for conditional activation of T3SS1.

Our goal was to develop a conditional system whereby extracellular *S*. Typhimurium would have a functional T3SS1 to allow for bacterial invasion but in which T3SS1 activity would be rapidly and perpetually disabled following mammalian cell entry. We considered the genes for four different T3SS1-associated proteins as potential targets for genetic manipulation: (i) HilE, a negative regulator of SPI1-encoded gene transcription (26, 27), which includes T3SS1 structural components and effectors, (ii) SipB and SipC, the two translocator proteins that form the T3SS translocon pore (28), and (iii) InvC, the energy-generating ATPase (29).

We first study a conditional mutant for which transcription of genes encoding structural components of T3SS1 and T3SS1-associated effectors could be modulated. HilE is the major negative regulator of SPI-1 gene expression. It interacts with HilD to inhibit transcription of HilA, a transcriptional activator that has a central role in modulating the expression of T3SS1 apparatus and T3SS1-associated effector genes (26). Overexpression of *hilE* represses *hilA* expression and drastically reduces bacterial invasion into nonphagocytic cells, whereas *hilE* mutants are derepressed for *hilA* transcription and exhibit a hyperinvasive phenotype (26) (Fig. S2A). We therefore reasoned that modulating *hilE* expression would allow us to temporally control T3SS1 activity. *hilE* under the control of the ATc-inducible *tetA* promoter (*P*_*tetA*_) (30) was inserted between *purA* and *yjeB-nsrR* in the chromosome of an *S*. Typhimurium Δ*hilE* mutant (Δ*hilE tetRA*-*hilE*). Addition of ATc to the bacterial subculture decreased the invasion efficiency (the percentage of the infective inoculum that was internalized at 1 h p.i.) of the Δ*hilE tetRA*-*hilE* strain to levels similar to those of a *prgI* mutant, indicating an SPI-1 deficiency (Fig. S2A). In the absence of ATc induction, the Δ*hilE tetRA*-*hilE* strain also showed decreased invasion compared to Δ*hilE* bacteria, suggesting leaky expression of *hilE*. Notably, intracellular induction of *hilE* expression with 300 ng/ml ATc, a concentration that is sufficient to induce gene expression in vacuolar and cytosolic *Salmonella* in epithelial cells (20), did not affect intracellular T3SS1 activity (SipC translocation) or bacterial proliferation. Specifically, the proportions of infected cells positive for translocated SipC (Fig. S2B) and vacuolar and cytosolic proliferation (Fig. S2C) were indistinguishable for cells infected with Δ*hilE tetRA*-*hilE* bacteria incubated in the presence or absence of ATc.

10.1128/mBio.01931-17.2 FIG S2  Controlled expression of *hilE* or *sipC* does not affect intracellular replication. (A) A cartoon depicting regulated *hilE* expression. *hilE* is under the control of *P*_*tetA*_ and was inserted in the chromosome of a Δ*hilE* mutant between *purA* (SL1344_4299) and *yjeB/nsrR* (SL1344_4300). The invasion efficiencies of *S*. Typhimurium WT, Δ*prgI* (a T3SS1 mutant), Δ*hilE*, and Δ*hilE tetRA*::*hilE* are shown Where indicated, subcultures were grown in the absence (gray) or presence (black) of 100 ng/ml ATc. The invasion efficiency (the percentage of inoculum internalized) was determined in a gentamicin protection assay in HeLa cells at 1 h p.i. and is expressed as a percentage of the invasion efficiency for the wild-type bacteria (set to 100%). (B) Time course of SipC delivery into epithelial cells. HeLa cells were infected with *S*. Typhimurium WT and Δ*hilE tetRA*-*hilE* (ATc-induced) bacteria (chromosomal *P_trc_-mCherry* strains). Where indicated (open squares), 300 ng/ml ATc was added to infected cells at 10 min p.i. and maintained throughout. Monolayers were fixed at 0.5 h, 4 h, and 8 h p.i. and immunostained for SipC. The percentage of infected cells positive for SipC signal was scored by fluorescence microscopy. (C) Single-cell analysis results of intracellular proliferation in epithelial cells. HeLa cells were infected as described for panel B and fixed at 1 h, 4 h, and 8 h p.i., and the number of bacteria in each infected cell was scored by fluorescence microscopy. Cells with ≥100 bacteria/cell contain cytosolic *S*. Typhimurium. Each dot represents 1 cell, and data from 3 independent experiments are shown (>180 cells in total). (D) Cartoon depicting regulated *sipB* and *sipC* expression. These genes are under the control of the *araBAD* promoter (*P*_*BAD*_) in the pBAD30 vector. Invasion efficiency of *S*. Typhimurium WT versus that in strains Δ*sipB* pBAD30, Δ*sipB* pBAD30-*sipB*, Δ*sipC* pBAD30, and Δ*sipC* pBAD30-*sipC*. Where indicated, subcultures were grown in the absence (gray) or presence (black) of 0.2% (wt/vol) arabinose. (E) Time course of SipC delivery into epithelial cells. Cells were infected with WT and Δ*sipC* pBAD30-*sipC* (arabinose-induced) bacteria (chromosomal *P_trc_-mCherry* strains). Arabinose (1% [wt/vol]) was added at *t*0 where indicated and maintained throughout. (F) Single-cell analysis of intracellular proliferation in epithelial cells. HeLa cells were infected as described for panel E. Each dot represents 1 cell, and data from 3 independent experiments are shown (>180 cells in total). For all panels, asterisks indicate results that were significantly different from those for the WT. Download FIG S2, PDF file, 2.5 MB. Copyright © 2017 Klein et al. 2017 Klein et al. This content is distributed under the terms of the Creative Commons Attribution 4.0 International license.

Next we attempted to conditionally control T3SS1 by placing *sipB* or *sipC* under the control of the *araBAD* (*P*_*BAD*_) promoter from *Escherichia coli* (31). We recently showed that the invasion defect of an *S*. Typhimurium Δ*sipB* or Δ*sipC* mutant could be complemented in *trans* with *sipB* or *sipC*, respectively (32), when under the control of this promoter. A similar arabinose-inducible system has been used previously to investigate the intracellular role of the *S. flexneri* translocator, IpaB (33), the SipB ortholog. To test the validity of arabinose-controllable expression of the T3SS1 translocator proteins, *S*. Typhimurium Δ*sipB* and Δ*sipC* mutants were transformed with pBAD30 (empty vector) and pBAD30-*sipB* or pBAD30-*sipC* constructs and tested for their ability to invade HeLa epithelial cells when grown in the presence or absence of arabinose. Overall, invasion efficiencies indicated that placing expression of *sipC*, but not of *sipB*, under the control of the *P*_*BAD*_ promoter is a viable option for tightly controlled, transient activation of T3SS1 activity (Fig. S2D). However, when we compared intracellular T3SS1 activity, while no SipC-positive cells were detected for Δ*sipC* pBAD30-*sipC* bacteria in the absence of arabinose at 4 h and 8 h p.i. (Fig. S2E), vacuolar and cytosolic replication were unaffected at 8 h p.i. (Fig. S2F).

**FIG 1:**
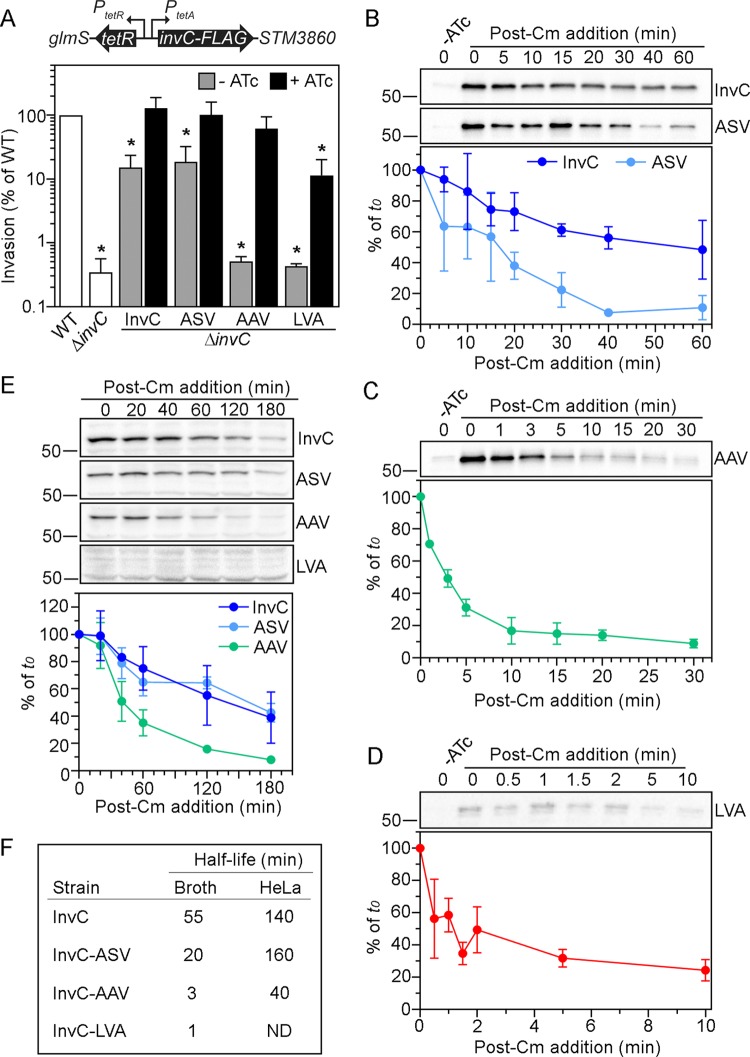
Progressive destabilization of InvC. (A) Cartoon depicting regulated *invC* expression. *invC* is under the control of *P*_*tetA*_ and was integrated by using a Tn*7* transposon at the site-specific *att*Tn*7*, located downstream of the *glmS* gene (SL1344_3828). Invasion efficiency of *S*. Typhimurium wild type (WT), Δ*invC*, Δ*invC tetRA-invC-*FLAG (InvC), Δ*invC tetRA-invC-*FLAG(ASV), Δ*invC tetRA-invC-*FLAG(AAV) and Δ*invC tetRA-invC-*FLAG(LVA). Where indicated, subcultures were grown in the absence or presence of 100 ng/ml ATc. Invasion efficiency (the percentage of inoculum internalized) was determined in a gentamicin protection assay in HeLa cells at 1 h p.i.; results are expressed as a percentage of the invasion efficiency for wild-type bacteria (set to 100%). Data are from ≥3 independent experiments (means ± SD). Asterisks indicate data significantly different from WT. (B, C, and D) InvC stability in broth. Δ*invC tetRA-invC-*FLAG (InvC), Δ*invC tetRA-invC-*FLAG(ASV), Δ*invC tetRA-invC-*FLAG(AAV), and Δ*invC tetRA-invC-*FLAG(LVA) bacterial subcultures were grown in the presence of 100 ng/ml ATc for 1 h to induce the synthesis of InvC-FLAG. ATc was washed out, and chloramphenicol (30 µg/ml) was added to stop *de novo* protein synthesis (*t*0). Aliquots were removed at the indicated times and processed for immunoblotting with anti-FLAG antibodies (α-FLAG). Similar exposure times were used for chemiluminescent detection. Graphs depict densitometric analysis results (means ± SD; 3 independent experiments). (E) InvC stability in HeLa epithelial cells. Bacterial strains (those shown in panel A) were grown in the presence of 100 ng/ml ATc for 1.5 h prior to infection to induce InvC-FLAG production. MOIs were adjusted so that equivalent numbers of bacteria were internalized into HeLa epithelial cells for all strains. At 30 min p.i., chloramphenicol (60 µg/ml) was added, then lysates were collected at the indicated times and processed for immunoblotting with anti-FLAG antibodies. Similar exposure times were used for chemiluminescence detection. The graph depicts densitometric analysis results (means ± SD; 3 independent experiments). (F) Summary of estimated half-lives for InvC-FLAG and its destabilized variants. ND, not determined.

SPI1 gene expression is downregulated inside cells, yet T3SS1 can remain functional for long periods if the apparatus is relatively stable once assembled. Such T3SS1 stability might explain why modulation of *hilE* and *sipC* expression did not have any overt effects on intracellular *Salmonella* (Fig. S2). We therefore tested a system in which T3SS1 activity was conditionally inactivated via manipulation of the stability of the T3SS1-energizing ATPase InvC. *invC* is essential for *Salmonella* invasion of nonphagocytic cells (34) and encodes the ATPase which energizes T3SS1 and facilitates unfolding of proteins for export (29, 34). Our rationale was that T3SS1 would be nonfunctional without ATP generation, i.e., it would be energetically “dead,” even if it remained assembled. We used Tn*7* transposon site-specific integration at the *att*Tn*7* locus, located downstream of the *glmS* gene (35), to chromosomally complement an *S*. Typhimurium *ΔinvC* mutant with *invC* under the control of *P*_*tetA*_, thereby allowing ATc-tunable *invC* expression. A 3× FLAG epitope was also attached to the carboxyl terminus of InvC to facilitate its immunodetection (Δ*invC glmS*::*tetRA-invC*-FLAG). To alter protein stability, we further incorporated one of three peptides recognized by bacterial tail-specific proteases—AANDENYA(ASV), AANDENYA(AAV), and AANDENYA(LVA) (36)—to the C terminus of InvC-FLAG. We tested the ability of these strains to enter HeLa epithelial cells when grown in the absence or presence of ATc. ATc induction fully restored invasion by Δ*invC glmS*::*tetRA-invC-*FLAG bacteria (Fig. 1A), indicating that 3× FLAG tag addition to the C terminus of InvC did not negatively affect its biological function and that ATc addition fully derepressed TetR binding to *P*_*tetA*_. However, these bacteria exhibited a reduced, yet significant, invasion efficiency in the absence of inducer, suggestive of leaky *invC* transcription under these growth conditions (Fig. 1A). Addition of C-terminal peptides to InvC differentially affected their ability to complement Δ*invC* bacteria for invasion: Δ*invC* bacteria producing InvC-FLAG(ASV) were the most competent for invasion when grown in the presence of ATc, and InvC-FLAG(LVA) was the least competent (Fig. 1A). Furthermore, under noninducing conditions, Δ*invC glmS*::*tetRA-invC-*FLAG(AAV) and Δ*invC glmS*::*tetRA-invC-*FLAG(LVA) strains exhibited invasion efficiencies equivalent to that of Δ*invC* bacteria (Fig. 1A), indicating negligible InvC function. Collectively, these results identified at least two strains for which InvC function is tightly controlled by ATc addition to bacterial subcultures [Δ*invC glmS*::*tetRA-invC-*FLAG(AAV) and Δ*invC glmS*::*tetRA-invC-*FLAG(LVA)], and these strains therefore are potential candidates for examining the intracellular effects of an inoperative T3SS1.

### InvC is destabilized by peptide tail addition.

The destabilizing effect of adding peptide tail variants has been assessed for green fluorescent protein (GFPmut3*) tagging in *E. coli* (36). Addition of these peptides to GFPmut3* dramatically shortens its half-life from >24 h to 110 min (ASV), 60 min (AAV), or 40 min (LVA) (36). Since our invasion data suggested that addition of each of these peptide tags differentially affected InvC turnover in *Salmonella* (Fig. 1A), we used a modified pulse-chase experiment to directly compare the half-lives of InvC-FLAG, InvC-FLAG(ASV), InvC-FLAG(AAV), and InvC-FLAG(LVA). For quantification of InvC variant half-lives, *invC* expression was induced by the addition of ATc to bacterial subcultures for 1 h. Bacteria were then washed to remove ATc and treated with chloramphenicol (time 0 [*t*0]) to prevent *de novo* protein synthesis. Bacterial lysates were collected over a time course and assessed for InvC-FLAG levels by immunoblotting (Fig. 1B, C, and D). We calculated the half-life of InvC-FLAG to be 55 min in *S*. Typhimurium; the addition of AANDENYA(ASV), AANDENYA(AAV), or AANDENYA(LVA) peptides to InvC reduced its half-life to 20 min, 3 min, and 1 min, respectively (Fig. 1B, C, D, and F). Therefore, these peptide sequences have a destabilizing effect on *S*. Typhimurium proteins, as observed for *E. coli*. Because we were aiming to rapidly inactivate T3SS1 energetics after bacterial internalization, we also compared the turnover of InvC and destabilized variants of InvC inside mammalian cells. HeLa epithelial cells were infected with ATc-induced bacterial subcultures, and at 30 min p.i., chloramphenicol was added (*t*0). Whole-cell lysates were collected at various time points, and InvC-FLAG levels were analyzed by immunoblotting (Fig. 1D). Interestingly, InvC-FLAG and its variants were more stable in intracellular *S*. Typhimurium than in liquid broth. InvC-FLAG and InvC-FLAG(ASV) had comparable half-lives for intracellular *S*. Typhimurium, 140 min and 160 min, respectively (Fig. 1E and F), whereas the half-life of InvC-FLAG(AAV) was considerably shorter (40 min) (Fig. 1E and F). We were unable to detect a band corresponding to the predicted size of InvC-FLAG(LVA) at *t*0 (30 min p.i.) (Fig. 1E), presumably due to its extremely rapid turnover, which precluded us from calculating its half-life. Our conservative estimate of the half-life of InvC-FLAG(LVA) is <10 min inside mammalian cells. Due to their accelerated turnover compared to InvC-FLAG, both InvC-FLAG(AAV) and InvC-FLAG(LVA) were further considered to effect inactivation of T3SS1 inside mammalian cells, a requirement for assessing its intracellular role. We also considered it advantageous to use multiple destabilized variants, since their differential stabilities allowed us to study the effects of “tuning” InvC production, akin to a dose-response curve for T3SS1 activity.

### T3SS1 activity is diminished for strains producing InvC-FLAG(AAV) and InvC-FLAG(LVA).

**FIG 2:**
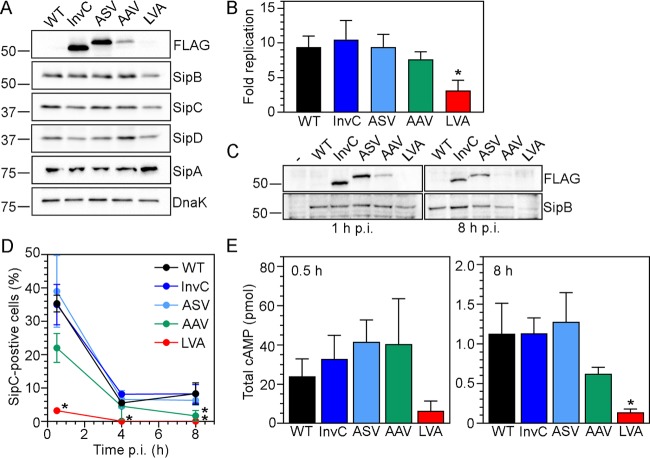
Altered InvC stability leads to tunable intracellular T3SS1 activity. (A) Immunoblotting detection of T3SS1 components in broth-grown bacteria. *S*. Typhimurium Δ*invC tetRA-invC-*FLAG (InvC), Δ*invC tetRA-invC-*FLAG(ASV), Δ*invC tetRA-invC-*FLAG(AAV), and Δ*invC tetRA-invC-*FLAG(LVA) subcultures were grown in the presence of 100 ng/ml ATc for 1.5 h to induce InvC-FLAG synthesis. Lysates from these strains and WT bacteria were probed with anti-FLAG (to detect InvC-FLAG), anti-SipB (T3SS1 translocator protein), anti-SipC (T3SS1 translocator protein), anti-SipD (T3SS1 tip complex protein), anti-SipA (T3SS1 effector), and anti-DnaK (loading control) antibodies. Blots are representative of two independent experiments. (B) Intracellular replication. Subcultures were grown as described for panel A and used to infect HeLa epithelial cells. MOIs were adjusted so that equivalent bacterial numbers were internalized for all strains. Monolayers were lysed for bacterial enumeration at 1 h and 8 h p.i. The fold replication over this timeframe is shown (mean ± SD; *n* ≥ 7 experiments). An asterisk denotes significantly different results from WT bacteria. (C) Immunoblotting detection of T3SS1 components during infection. HeLa cells were infected as described for panel B, and monolayers were collected at 1 h and 8 h p.i. and subjected to immunoblotting with anti-FLAG and anti-SipB antibodies. Samples were standardized to an equal number of bacteria for each time point. Blots are representative of three independent experiments. (D) Time course of SipC delivery into epithelial cells. HeLa cells were infected with *S*. Typhimurium WT and ATc-induced bacterial subcultures (harboring pFPV*-*mCherry) as described for panel B. Monolayers were fixed at 0.5 h, 4 h, and 8 h p.i. and immunostained for SipC. The percentage of infected cells positive for SipC signal was scored by fluorescence microscopy. Asterisks indicate data significantly different from WT. (E) SopB-CyaA translocation. HeLa epithelial cells were infected as described for panel B with strains harboring a SopB-CyaA plasmid. Lysates were collected at 0.5 h and 8 h p.i. and subjected to an ELISA for cAMP quantification (total cAMP/well). For reference, cAMP levels in WT bacteria-infected lysates (i.e., no SopB-CyaA plasmid) were 0.24 ± 0.15 pmol/well and 0.10 ± 0.044 pmol/well at 0.5 h and 8 h p.i., respectively. An asterisk denotes a result significantly different from that of WT SopB-CyaA bacteria.

To examine the effect of InvC destabilization on T3SS1, we screened for the temporal abundance of T3SS1 components in liquid broth and infected cells. Whole-cell lysates from bacterial subcultures (induced with ATc where appropriate) were subject to immunoblotting with anti-FLAG, anti-SipB, anti-SipC, anti-SipD, anti-SipA, and anti-DnaK (bacterial loading control) antibodies. In agreement with the calculated half-lives of the InvC variants (Fig. 1F), the steady-state levels of InvC-FLAG decreased as the stability decreased (Fig. 2A). Levels of SipB, SipC, SipD, and SipA were comparable for all tested strains (Fig. 2A), indicating that there was no feedback response on the stability of other T3SS1 components or T3SS1-associated effectors for broth-grown bacteria.

The ability of the InvC variant strains to replicate in HeLa epithelial cells was assessed in a gentamicin protection assay. Multiplicities of infection (MOIs) were adjusted so that equivalent numbers of bacteria were internalized. From 1 h to 8 h p.i., SL1344 wild type, Δ*invC glmS*::*tetRA-invC-*FLAG(ASV), and Δ*invC glmS*::*tetRA-invC-*FLAG(AAV) strains had equivalent levels of replication: 9.4-fold, 10.5-fold, and 9.4-fold, respectively (Fig. 2B), but Δ*invC glmS*::*tetRA-invC-*FLAG(ASV) (7.7-fold) and *glmS*::*tetRA-invC-*FLAG(LVA) bacteria (3.1-fold) had decreased levels of replication (Fig. 2B). Whole-cell lysates were also collected at 1 h and 8 h p.i. and subjected to immunoblotting with anti-FLAG and anti-SipB antibodies. Loading was normalized for equivalent CFU per lane for each time point. There was a progressive reduction in intracellular InvC-FLAG levels at both 1 h and 8 h p.i., as protein stability decreased (Fig. 2C), i.e., the temporal profiles for InvC-FLAG and InvC-FLAG(ASV) were equivalent, whereas InvC-FLAG(AAV) and InvC-FLAG(LVA) were moderately and drastically reduced, respectively. Intracellular SipB levels for all strains were equivalent to those in wild-type bacteria at 1 h p.i. and 8 h p.i., except for Δ*invC glmS*::*tetRA-invC-*FLAG(LVA) bacteria, which had considerably lower levels at 8 h p.i. than observed for all other strains (Fig. 2C). Collectively, these data indicate that, at later times during infection, InvC abundance is affected for the InvC-FLAG(ASV) strain and for both InvC and SipB abundance levels for the InvC-FLAG(LVA) strain.

We then compared intracellular T3SS1 activity for these strains via immunostaining and adenylate cyclase (CyaA) assays. For immunostaining, bacteria harbored pFPV-mCherry, a plasmid that carries *mCherry* under the control of the constitutive *S*. Typhimurium *rpsM* promoter (37), to facilitate fluorescence detection. Subcultures were induced with ATc where appropriate, and infected HeLa cells were processed for SipC immunodetection at 30 min, 4 h, and 8 h p.i. SipC is a T3SS1 translocator protein and type III effector, and we recently showed that SipC localizes to numerous puncta on the surface of, and in the vicinity of, bacteria as early as 15 min p.i. (32). The temporal decrease in the proportion of SipC-positive infected cells was indistinguishable for SL1344 wild type, Δ*invC glmS*::*tetRA-invC-*FLAG, and Δ*invC glmS*::*tetRA-invC-*FLAG(ASV) bacteria (Fig. 2D), whereas significantly fewer SipC-positive infected cells were detected at 8 h p.i. for Δ*invC glmS*::*tetRA-invC-*FLAG(AAV) bacteria and at all time points for Δ*invC glmS*::*tetRA-invC-*FLAG(LVA) bacteria (Fig. 2D). Using a CyaA reporter system (38), we also directly quantified T3SS1-mediated translocation of SopB/SigD, a type III effector that is required for efficient *S*. Typhimurium invasion of epithelial cells (39) and continues to be synthesized and translocated for many hours after bacterial internalization (23, 40–42). HeLa epithelial cells were infected with *S*. Typhimurium strains harboring SopB-CyaA. MOIs were adjusted so that equivalent numbers of bacteria were internalized, and cAMP was quantified at 30 min and 8 h p.i. in an enzyme-linked immunosorbent assay (ELISA). Infection with wild type, Δ*invC glmS*::*tetRA-invC-*FLAG, Δ*invC glmS*::*tetRA-invC-*FLAG(ASV), or Δ*invC glmS*::*tetRA-invC-*FLAG(AAV) bacteria led to equivalent levels of cAMP production at 0.5 h p.i., whereas SopB-CyaA translocation by Δ*invC glmS*::*tetRA-invC-*FLAG(LVA) bacteria was considerably less (Fig. 2E). By 8 h p.i., cAMP levels in infected lysates were ≥20-fold less for all strains (Fig. 2E). Equivalent cAMP was detected in lysates from wild type, Δ*invC glmS*::*tetRA-invC-*FLAG, and Δ*invC glmS*::*tetRA-invC-*FLAG(ASV) infections at 8 h p.i., whereas less cAMP was detected for Δ*invC glmS*::*tetRA-invC-*FLAG(AAV) bacteria (Fig. 2E). Lysates from cells infected with Δ*invC glmS*::*tetRA-invC-*FLAG(LVA) bacteria exhibited only background cAMP levels (SL1344 wild type with no plasmid) at 8 h p.i. (Fig. 2E). Taken together, our results indicate that these destabilized InvC variants yield *S*. Typhimurium strains with a gradation of T3SS1 activity; bacteria with InvC-FLAG(ASV) have “high” T3SS1 activity that is comparable to that of wild-type and InvC-FLAG bacteria, whereas those with InvC-FLAG(AAV) and InvC-FLAG(LVA) have “medium” and “low” T3SS1 activity, respectively.

### Identification of intracellular activities associated with T3SS1 activity.

**FIG 3:**
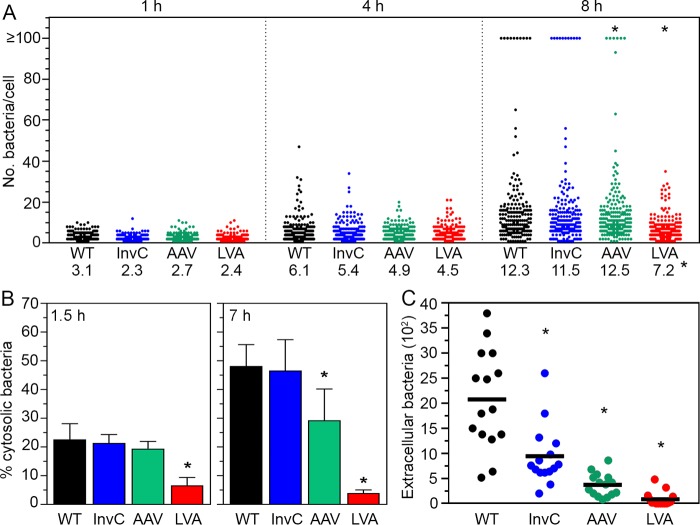
Identification of postinvasion activities of T3SS1. (A) Single-cell analysis of intracellular proliferation. *S*. Typhimurium Δ*invC tetRA-invC-*FLAG (InvC), Δ*invC tetRA-invC-*FLAG(AAV), and Δ*invC tetRA-invC-*FLAG(LVA) bacterial subcultures were grown in the presence of 100 ng/ml ATc for 1.5 h prior to infection to induce the synthesis of InvC-FLAG. HeLa epithelial cells were infected with the above strains and WT bacteria (harboring pFPV-mCherry). MOIs were adjusted so that equivalent numbers of bacteria were internalized. Monolayers were fixed at 1 h, 4 h, and 8 h p.i., and the number of bacteria in each infected cell was blindly scored by fluorescence microscopy. Cells with ≥100 bacteria contained cytosolic *S*. Typhimurium. Each dot represents one infected cell, and data from 3 independent experiments are shown (>180 cells in total). Values at the bottom of the panel indicate the mean number of bacteria per cell for cells containing <99 bacteria/cell (i.e., cells with ≥100 bacteria were excluded from the statistical analysis). Asterisks at the top and bottom of the panel indicate cytosolic and vacuolar replication that were significantly different from WT bacteria, respectively. (B) CHQ resistance assay. Subcultures were grown as described for panel A and used to infect HeLa epithelial cells. The proportion of cytosolic bacteria was determined at 1.5 h and 7 h p.i. in a CHQ resistance assay. Asterisks denote results that were significantly different from those of WT bacteria. (C) Bacterial egress. HeLa epithelial cells were infected as described for panel B. At 7 h p.i., gentamicin-containing growth medium was replaced with gentamicin-free medium for 1 h, and the number of extracellular bacteria was determined by agar plating. Each point is from an individual well in a 24-well tissue culture plate, and data are from 5 independent experiments. Means are indicated by horizontal lines, and asterisks denote significantly different results from those with WT bacteria.

We used these strains with tunable T3SS1 activity—InvC-FLAG, InvC-FLAG(AAV) and InvC-FLAG(LVA)—to dissect the contribution of T3SS1 to specific stages in the intracellular lifecycle of *Salmonella*. We precluded the InvC-FLAG(ASV) strain from further analysis because it was indistinguishable from the InvC-FLAG strain in all of the preceding assays (Fig. 2 and 3). We first used single-cell analysis to monitor bacterial proliferation in epithelial cells by fluorescence microscopy. *S*. Typhimurium strains carried pFPV-mCherry, and MOIs were adjusted so that equivalent numbers of bacteria were internalized into HeLa epithelial cells. At 1 h p.i., the number of bacteria/cell was comparable for all *S*. Typhimurium strains, ranging from an average of 2.3 to 3.1 bacteria/cell (Fig. 3A). Wild-type and Δ*invC glmS*::*tetRA-invC-*FLAG bacteria showed similar vacuolar replication at 4 h and 8 h p.i. (Fig. 3A), respectively, and also for the proportion of infected cells containing cytosolic bacteria (≥100 bacteria) (20, 43, 44) at 8 h p.i. (6.6% ± 2.4% and 6.3% ± 2.0%) (data are means ± standard deviations [SD]) (Fig. 3A), in agreement with the results from the gentamicin protection assays (Fig. 2B). While vacuolar replication for Δ*invC glmS*::*tetRA-invC-*FLAG(AAV) bacteria was unaffected, the proportion of cells with cytosolic bacteria was significantly reduced at 8 h p.i. (3.3% ± 1.2%) (Fig. 3A). For Δ*invC glmS*::*tetRA-invC-*FLAG(LVA) bacteria, only 0.26% ± 0.58% of infected HeLa cells contained cytosolic *Salmonella* at 8 h p.i., and the mean number of vacuolar bacteria/cell was also significantly reduced (Fig. 3A), indicating compromised cytosolic and vacuolar proliferation for this strain, consistent with the gentamicin protection assay results (Fig. 2B).

We next used a population-based assay to quantify the proportion of the total bacterial population residing in the cytosol. The chloroquine (CHQ) resistance assay relies on the lysosomotropic properties of CHQ, which accumulates only within the SCV and leads to the killing of vacuolar *S*. Typhimurium by an as-yet-unidentified mechanism but leaves the cytosolic population unharmed. In conjunction with a gentamicin protection assay, this determines the percentage of internalized bacteria that populate the cytosol at a given time (20). HeLa cells were infected with *S*. Typhimurium wild type, Δ*invC glmS*::*tetRA-invC-*FLAG, Δ*invC glmS*::*tetRA-invC-*FLAG(AAV), and Δ*invC glmS*::*tetRA-invC-*FLAG(LVA) bacteria such that equivalent numbers were initially internalized, and the proportion of cytosolic bacteria was determined at 1.5 h and 7 h p.i. At 1.5 h p.i., this proportion was equivalent for *S*. Typhimurium wild type, Δ*invC glmS*::*tetRA-invC-*FLAG, and Δ*invC glmS*::*tetRA-invC-*FLAG(AAV) bacteria, whereas it was significantly reduced for Δ*invC glmS*::*tetRA-invC-*FLAG(LVA) bacteria (Fig. 3B). By 7 h p.i., the phenotype of Δ*invC glmS*::*tetRA-invC-*FLAG(LVA) bacteria was much more dramatic, with only 3.9% ± 1.1% of the total population present in the cytosol, compared to 46% ± 5.7% and 47% ± 11% for wild-type and Δ*invC glmS*::*tetRA-invC-*FLAG bacteria, respectively (Fig. 3B). The Δ*invC glmS*::*tetRA-invC-*FLAG(AAV) bacteria were also defective for cytosolic colonization at 7 h p.i. (29% ± 11%) (Fig. 3B), which is consistent with the single-cell analysis results (Fig. 3A). Importantly, the cytosolic colonization defects for InvC-FLAG(AAV) and InvC-FLAG(LVA) strains were recapitulated in other epithelial cell lines (Fig. S1). Altogether, these results uniformly demonstrated that bacteria producing InvC-FLAG(LVA), the most destabilized variant of InvC, are prominently defective for lysis of the nascent vacuole, which subsequently affects the frequency of cytosolic colonization. In contrast, bacteria producing InvC-FLAG(AAV), which is of intermediate stability, are not affected for nascent SCV lysis or vacuolar replication but are compromised for cytosolic growth at later times. This identifies a novel contribution of intracellular T3SS1 activity to the cytosolic proliferation of *Salmonella* which is distinct from its previously characterized role in nascent vacuole lysis and trafficking.

Finally, we tested the effects of destabilizing InvC on bacterial egress as a potential consequence of cytosolic replication and epithelial cell lysis (19). If cytosolic bacteria are the intracellular population that seed reinfection, we predicted a direct correlation between the number of cytosolic bacteria and the efficiency of their egress. To test this, HeLa epithelial cells were infected with the same strains as for the CHQ resistance assay (adjusting MOIs so that equivalent numbers of bacteria were internalized), and at 7 h p.i., gentamicin-containing medium was removed and replaced with gentamicin-free medium for 1 h, after which the number of extracellular bacteria was enumerated by agar plating. There was a progressive decrease in bacterial egress that correlated with InvC stability (Fig. 1F) and the proportion of cytosolic bacteria (Fig. 3A and B): InvC-FLAG > InvC-FLAG(ASV) > InvC-FLAG(LVA) (Fig. 3C). This specifically implicated cytosolic bacteria as the intracellular subset that is released from epithelial cells. Furthermore, all InvC-FLAG variants were affected for bacterial release compared to wild-type bacteria (Fig. 3C), even InvC-FLAG, which is indistinguishable from wild-type bacteria with respect to vacuolar and cytosolic replication at late times postinfection (Fig. 3A and B). Because the InvC-FLAG strain is not capable of reinducing T3SS1 activity in the absence of the ATc inducer, this also identifies resurgent T3SS1 activity as being requisite for bacterial egress.

### SopB and SipA are required for efficient cytosolic proliferation by *Salmonella*.

**FIG 4:**
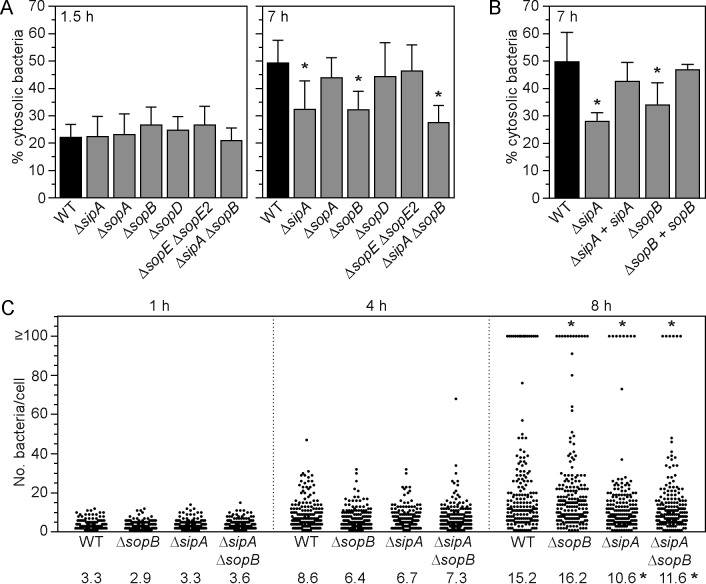
SopB and SipA are required for efficient cytosolic replication. (A) HeLa epithelial cells were infected with *S*. Typhimurium WT and the indicated T3SS1 effector deletion mutants, and the proportions of cytosolic bacteria at 1.5 h and 7 h p.i. were determined in a CHQ resistance assay. Asterisks denote data significantly different from WT bacteria. (B) Complementation analysis. HeLa cells were infected with *S*. Typhimurium WT or Δ*sipA* or Δ*sopB* deletion mutants and these deletion mutants complemented with a single gene copy via Tn*7* integration at the *att*Tn*7* site. The proportion of cytosolic bacteria at 7 h p.i. was determined using the CHQ resistance assay. Asterisks indicate results significantly different from those for WT bacteria. (C) Single-cell analysis of intracellular proliferation. HeLa epithelial cells were infected with *S*. Typhimurium WT, Δ*sopB*, Δ*sipA*, and Δ*sipA* Δ*sopB* bacteria (harboring pFPV-mCherry). Monolayers were fixed at 1 h, 4 h, and 8 h p.i., and the number of bacteria in each infected cell was blindly scored by fluorescence microscopy. Cells with ≥100 bacteria contain cytosolic *S*. Typhimurium. Each dot represents one infected cell, and data from 3 independent experiments are shown (>180 cells in total). Values at the bottom of the panel indicate the mean number of bacteria per cell for cells containing <99 bacteria/cell (i.e., cells with ≥100 bacteria were excluded from the statistical analysis). Asterisks at the top and bottom of the panel indicate cytosolic and vacuolar replication, respectively, that was significantly different from that of WT bacteria.

Our results designated a newly appreciated role for intracellular T3SS1 activity with regard to cytosolic proliferation of *Salmonella*. T3SS1 effectors can be detected at later times (≥8 h p.i.) in epithelial cells, albeit to a lesser extent than shortly after initial bacterial entry (Fig. 2) (23, 40–42, 45). To investigate whether any of the known T3SS1 effectors are involved in bacterial colonization of the epithelial cell cytosol, HeLa cells were infected with *S*. Typhimurium wild-type, Δ*sipA*, Δ*sopA*, Δ*sopB*, Δ*sopD*, or Δ*sopE* Δ*sopE2* bacteria, and the CHQ resistance assay was used to quantify the proportion of cytosolic bacteria in the total populations at 1.5 h and 7 h p.i. Compared to wild-type bacteria, no effector deletion mutants were compromised for nascent vacuole lysis at 1.5 h p.i., but two mutants had a significantly reduced proportion of cytosolic bacteria at 7 h p.i.: Δ*sipA* and Δ*sopB* (Fig. 4A). Notably, this defect was restored to wild type levels upon complementation with a single gene copy in the respective mutant (Fig. 4B). A Δ*sipA* Δ*sopB* double mutant was not more affected than either single deletion mutant at 7 h p.i. (Fig. 4A). Single-cell analysis corroborated that Δ*sipA*, Δ*sopB* and Δ*sipA* Δ*sopB* mutants were all affected for the proportion of infected cells containing cytosolic bacteria (≥100 bacteria/cell; 9.5 ± 1.5% for wild type, 6.0 ± 1.6% for Δ*sopB*, 4.0 ± 0.93% for Δ*sipA*, and 3.0 ± 1.6% for Δ*sipA* Δ*sopB* bacteria) (Fig. 4C). Δ*sipA* and Δ*sipA* Δ*sopB* mutants were also compromised for replication in the SCV (Fig. 4C). Altogether, these data implicate the activities of SipA and SopB, two T3SS1-dependent effectors, as being required for the efficient cytosolic proliferation of *Salmonella*, likely via a common or converging mechanism.

## DISCUSSION

Here, we have used a number of different approaches to affect T3SS1 inactivation dynamics, and in order to analyze the intracellular contributions of this invasion-associated injectisome to *S*. Typhimurium. One approach we chose was an inducible expression system whereby the conditional mutant acts like wild-type bacteria when in the presence of an inducer (extracellularly) and ideally like a T3SS1 mutant in the absence of inducer (inside mammalian cells). A similar tactic has been used before to investigate the postinvasion roles of the *Shigella* T3SS. By placing various *S. flexneri* genes encoding T3SS structural components under the control of an arabinose-inducible promoter, two studies have demonstrated that the *S. flexneri* Mxi-Spa T3SS is required for different aspects of cell-to-cell spread (33, 46). Recently, Finn et al. (23) employed arabinose-inducible expression of *S*. Typhimurium *invA*, which encodes a T3SS1 structural protein, to generate a T3SS1-defective mutant in the intracellular environment and showed that this strain was compromised for SopB and SipA translocation into HeLa cells, but not in Caco-2 C2BBe1 cells, specifically by cytosolic *S*. Typhimurium. Inducible gene expression has also been exploited to define the temporal contribution of the T4SS to intracellular colonization. Using isopropyl-β-d-thiogalactopyranoside (IPTG)-inducible expression of *dotA*, which encodes a *Legionella pneumophila* Dot/Icm T4SS structural component, Roy et al. showed that DotA was required for intracellular replication of *L. pneumophila*, but only at time points after 24 h p.i. (47). Similar conclusions were drawn using a Cre/*loxP in vivo* gene deletion system in *L. pneumophila* (48). Most recently, via ATc-regulatable expression of the *Brucella abortus* T4SS ATPase VirB11, Smith et al*.* demonstrated that bacterial egress (at ~72 h p.i.) was dependent on the VirB T4SS (49). The premise of the inducible expression approach used in all these studies is that, in the absence of gene expression, there is sufficient protein turnover to intracellularly inactivate the secretion system being studied. However, several lines of evidence suggest this is not necessarily so for intracellular bacteria. For example, *L. pneumophila* IcmQ, a Dot/Icm T4SS structural protein, appears to be an extremely stable protein in infected macrophages (48). Furthermore, the half-life of the *B. abortus* VirB11 ATPase is considerably extended inside macrophages (80 min) compared to that in liquid broth (16 min) (49). Similarly, we have shown that the half-life of *S*. Typhimurium InvC, the ATPase for the invasion-associated injectisome, is increased when bacteria are inside epithelial cells (140 min) compared to bacteria in broth culture (55 min) (Fig. 1). Not all T3SS structural proteins are long-lived inside mammalian cells, however. While the half-life of SipC, one of the T3SS1 translocator proteins, is <10 min for broth-grown *S*. Typhimurium (50), it is ~25 min inside epithelial cells (Fig. S3). SipB, the other T3SS1 translocator protein, and SipD, the T3SS1 tip complex protein, similarly have short half-lives in mammalian cells (Fig. S3). Altogether, these findings suggest that the T3SS basal body is a more stable structure than the T3SS needle-associated complexes. With such short half-lives, SipB, SipC, or SipD would seem logical targets to manipulate intracellular T3SS1 activity. However, the Δ*sipC* pBAD30-*sipC* strain lacked a phenotype in HeLa epithelial cells upon arabinose withdrawal (Fig. S2B and C). This result could be interpreted as showing that T3SS1 has little impact upon the intracellular colonization of *Salmonella*. However, it was recently shown that SopB and SipA delivery by cytosolic *Salmonella* are independent of SipB (23). Taken together, we favor the explanation that T3SS1 delivery of effectors by cytosolic *Salmonella* is independent of SipB and SipC translocator proteins, perhaps more akin to a contact-independent secretion-like process that is triggered in broth culture.

10.1128/mBio.01931-17.3 FIG S3  SipB, SipC, and SipD have short half-lives in mammalian cells. HeLa epithelial cells were infected with *S*. Typhimurium SL1344 sipB::3× FLAG, sipC::3× FLAG, or sipD::3× FLAG strains, and at 30 min p.i. chloramphenicol (60 µg/ml) was added to stop *de novo* protein synthesis. Lysates were collected at the indicated times and subjected to immunoblotting with anti-FLAG antibodies. The graph depicts densitometric analysis results from 3 independent experiments. Download FIG S3, PDF file, 0.6 MB. Copyright © 2017 Klein et al. 2017 Klein et al. This content is distributed under the terms of the Creative Commons Attribution 4.0 International license.

We have overcome the technical limitation imposed by protein stability by the addition of C-terminal destabilizing peptide tags. By using tags that confer different protein stability, we can control how long the InvC ATPase persists and thereby “tune” intracellular T3SS1 activity. For *S*. Typhimurium producing the most destabilized version of InvC, InvC(LVA), we saw the most dramatic intracellular phenotypes. Compared to wild-type bacteria, this strain was defective for the translocation of SipC and SopB (Fig. 2), nascent vacuole lysis (Fig. 3), and cytosolic and vacuolar replication and bacterial egress (Fig. 3). Studies using surrogate-mediated entry of T3SS1 null mutants (cointernalization with wild-type bacteria or invasin-mediated entry) have led to the idea that T3SS1 damages some nascent vacuoles, which leads to the cytosolic access of bacteria within compromised SCVs, followed by their autophagy-dependent elimination or autophagy-promoted repair of the damaged SCV (21, 51). Our findings with this T3SS1 “low-activity” mutant further advocate for the level of T3SS1 activity influencing the extent of nascent vacuole lysis. Upon invasin-mediated entry of an *S*. Typhimurium Δ*invA* mutant into HeLa epithelial cells, this T3SS1 null mutant is defective for intracellular replication at 6 h p.i. compared to wild-type bacteria (25); whether this is due to a replication defect in the vacuole and/or cytosol was not investigated. Our T3SS1 low-activity strain clarifies that bacterial replication within both intracellular niches is T3SS1-dependent (Fig. 3A). *S*. Typhimurium with the intermediate destabilized InvC, InvC(AAV), was only affected for later intracellular events, i.e., cytosolic proliferation and bacterial egress (Fig. 3), which allowed us to assign a role for T3SS1 in the cytosolic proliferation of *Salmonella* that is independent of defects in nascent vacuole lysis and trafficking.

After internalization of *S*. Typhimurium into nonphagocytic cells, SPI1 gene expression and T3SS1 activity are downregulated (13, 14). While numerous environmental cues have been identified that activate T3SS1, comparatively little is known about what down-modulates its activity, or how its function is terminated, once bacteria are inside mammalian cells. One study showed that the downregulation of *hilA* and *hilD* gene expression after bacterial entry into epithelial cells is retarded for an *S*. Typhimurium Δ*lon* mutant (13). In support of this, HilC and HilD are stabilized in a Δ*lon* mutant (52), although an independent study found only a minor effect of Lon on HilD stability (53). Therefore, whether Lon protease is involved in the postinvasion degradation of transcriptional regulators of SPI1 gene expression remains an open question. Interestingly, while HilE has been shown to negatively affect *hilA* transcription *in vivo* (26), a Δ*hilE* mutant is comparable to wild-type *S*. Typhimurium for the timing and extent of *hilA* and *hilD* downregulation in epithelial cells (13). This suggests that HilE is not responsible for turning off SPI1 gene expression intracellularly.

The InvC-FLAG strain, which is indistinguishable from wild-type bacteria with regard to the kinetics of downregulation of intracellular T3SS1 activity (Fig. 2) and load of cytosolic bacteria at later times (Fig. 3), is defective for bacterial release from epithelial cells. Since this strain is unable to reinduce intracellular T3SS1 activity in the absence of ATc, this suggests that resurgent T3SS1 activity is required for efficient bacterial egress (Fig. 3). While little is known about the conditions within the intracellular microenvironment that trigger the cessation of SPI1 gene expression, even less is known about how *Salmonella* reactivates SPI1 gene expression (19, 20, 23) and T3SS1 activity (Fig. 2) (23) once it reaches the epithelial cell cytosol. For example, is T3SS1 degraded in vacuolar bacteria or diluted by bacterial growth in the vacuole and then new T3SS1 complexes are synthesized once *Salmonella* has reached the cytosol? This would seem to be very energetically costly to a bacterium. A more likely scenario is that T3SS1 remains intact but T3SS1 type III effectors are no longer synthesized, or translocation of T3SS1-dependent effectors is somehow prevented and then reengaged when required. Since many bacteria rely on secretion systems to deliver effectors at specific doses and times during their infectious cycle, understanding how bacteria control the stability, turnover, and dynamics of these nanomachines are important questions to address in the future (5).

We recently identified three *S*. Typhimurium genes, *corA*, *recA*, and *ydgT*, that are required for efficient bacterial colonization of the epithelial cell cytosol (44). Here, we identified two more cytosolic colonization factors, SipA and SopB (Fig. 4). Unlike what we observed with the Δ*sopB* mutant (Fig. 4), Finn et al. found no difference between cytosolic replication of wild-type and Δ*sopB* bacteria in HeLa cells (23); the reason for this discrepancy remains unknown. Both SopB and SipA are type III effectors that continue to be synthesized and translocated by T3SS1 for many hours after bacterial internalization (23, 40–42, 45). In the case of SopB, this type III effector localizes to the plasma membrane and nascent SCV shortly after bacterial internalization (40, 41, 54), on mature SCVs (40), and unidentified structures in cells containing cytosolic *Salmonella* cells (23). SopB is known to activate the mammalian prosurvival kinase Akt and thereby delay the onset of apoptosis in epithelial cells (55). Recently SopB was shown to specifically delay the onset of cell death in epithelial cells containing cytosolic *Salmonella* (23), which occurs by pyroptosis (56). It is not known how SopB might delay epithelial cell pyroptosis or whether the reported SopB-dependent 70-min delay in cell death requires Akt activation, as no direct connection between Akt and pyroptosis has been established. SipA is an actin-binding protein that promotes actin polymerization (57–61) and accumulates as discrete foci near bacteria shortly after their internalization (62, 63), on the mature SCV (45), and as undefined puncta in cells harboring cytosolic *Salmonella* (23). As assessed in the gentamicin protection assay, a *sipA* deletion mutant is attenuated for total bacterial replication in NIH 3T3 fibroblasts (45). We have shown that SipA contributes to proficient replication in both the SCV and cytosol at later times (Fig. 4). This poses the question of how the activity of SipA contributes to bacterial proliferation in such distinct intracellular niches. SipA might alter membrane stability of the mature SCV due to changes in actin accumulation, as vacuoles surrounding Δ*sipA* and *sipA*^++^ bacteria (*sipA*^++^ bacteria express enhanced levels of SipA) are associated with poor and enriched F-actin recruitment at 6 h p.i. in fibroblasts, respectively, and *sipA*^++^ SCVs are inherently unstable (45).

In summary, here we have shown that newly appreciated key stages in the infection cycle of *Salmonella*, lysis of the internalization vacuole and subsequent hyperreplication in the cytosol of epithelial cells, are dependent on T3SS1 activity. While T3SS1 was originally designated the “invasion-associated injectisome,” based on its absolute requirement for directing bacterial entry into nonphagocytic cells, cumulative work from others and us emphasizes that this designation is no longer all-encompassing. We envision that disengaging the activity of the secretion machinery by targeting the energy-providing ATPase will prove to be a useful strategy for pinpointing the temporal contributions of secretion systems to the infectious cycle of other pathogenic bacteria.

## MATERIALS AND METHODS

### Bacterial strains and plasmid construction.

*Salmonella enterica* serovar Typhimurium SL1344 was the wild-type strain used in this study (64). The SL1344 Δ*sopB*, Δ*sopE* Δ*sopE2*, Δ*sipB*::FRT, and Δ*sipC*::FRT mutants have been described previously (8, 65, 66). SL1344 strains bearing chromosomal 3× FLAG-tagged SipB, SipC, and SipD have also been described (67). The *S*. Typhimurium Δ*prgI*::FRT mutant was constructed by excision of the kanamycin resistance cassette from Δ*prgI*::kan (20) by using pCP20 (68). Unmarked in-frame deletions of *invC* (Δ*invC*, with deletion of amino acids 6 to 410) and *sopA* (Δ*sopA*, with deletion of amino acids 11 to 773) were constructed in *S*. Typhimurium SL1344 by *sacB*-mediated allelic exchange as we have described previously (44). The SL1344 Δ*sipA*::kan and Δ*sopD*::kan mutants were constructed in *S*. Typhimurium SL1344 by using λ-Red recombinase technology (68) with the oligonucleotides sipA-KO-F/sipA-KO-R, and sopD-KO-F/sopD-KO-R, respectively (primer sequences are listed in Table S1). The SL1344 Δ*sopB* Δ*sipA*::kan strain was created by P22 transduction from Δ*sipA*::kan into Δ*sopB* and the SL1344 Δ*sipA*::FRT mutant by excision of the Kan^r^ cassette from Δ*sipA*::kan bacteria by using pCP20.

10.1128/mBio.01931-17.4 TABLE S1  Oligonucleotides used for cloning. Download TABLE S1, DOCX file, 0.2 MB. Copyright © 2017 Klein et al. 2017 Klein et al. This content is distributed under the terms of the Creative Commons Attribution 4.0 International license.

The Δ*sopB* mutant was complemented by Tn*7* integration of *sopB-sigE* at the *att*Tn*7* site. The *sopB-sigE* coding regions (*sigE* encodes the cognate chaperone of SopB) and 414 bp of upstream region were amplified from *S*. Typhimurium SL1344 genomic DNA with Xho-SopBcomp-F and Sma-SigEcomp-R. The amplicon was digested with XhoI/SmaI and ligated into XhoI-SmaI-digested pGP-Tn*7*-Cm (Amp^r^ Cm^r^) (35). The pGP-Tn*7*-*sopB-sigE* construct was transferred to *E. coli* SM10λpir cells and conjugated into the *S*. Typhimurium Δ*sopB* strain bearing pSTNSK, which carries the Tn*7* transposase-encoding genes *tnsABCD* (35). Chloramphenicol-resistant *S*. Typhimurium colonies were screened for the presence of *sopB-sigE* by PCR with *sopB*-specific primers and primers flanking *glmS* and *sl3827* (35). A similar strategy was used to complement the Δ*sipA*::FRT deletion mutant. The *sicA* promoter (*sipA* is transcribed as part of the *sicA-sipBCDA* operon) was amplified from SL1344 genomic DNA with XhoI-SipAcomp-F and SipACyaA-OLR, and the *sipA* open reading frame was amplified with SipA-CyaA-OLF and SmaI-SipAcomp-R. These two amplicons were mixed and reamplified with XhoI-SipAcomp-F and SmaI-SipAcomp-R, followed by digestion with XhoI/SmaI and ligation into XhoI/SmaI-digested pGP-Tn*7*-Cm to create pGP-Tn*7*-Cm-*sipA*. This construct was used to insert *sipA* at the *att*Tn*7* site located downstream of the *glmS* gene in the SL1344 Δ*sipA*::FRT chromosome.

Construction of FLAG-tagged InvC variants in *S*. Typhimurium was done in a stepwise fashion. First, the *tetR*-*P*_*tetA*_ region (including the *tetA* ribosome-binding site [RBS]) was amplified from pJC45 (69) using Kpn-tetR-R and tetARBS-invC-R, and the *invC* coding sequence was amplified from *S*. Typhimurium SL1344 genomic DNA by using tetARBS-invC-F and XhoI-InvC-R. These two amplicons were mixed and reamplified with Kpn-tetR-R and XhoI-invC-R, followed by digestion with KpnI/XhoI and ligation into KpnI/XhoI-digested pGPTn*7*-Cm to create pGPTn*7*-Cm-invC. This plasmid was used as a template for amplification with Kpn-tetR-R and XhoI-invCFLAG-R, and a similar cloning protocol was used to create pGPTn*7*-Cm-invCFLAG. Finally, this plasmid was used as a template with Kpn-tetR-R and one of three primers (XhoI-FLAGLVA-R, XhoI-FLAGAAV-R, or XhoI-FLAGASV-R) to amplify *invC-FLAG* variants with destabilizing C-terminal peptide tags (36), pGPTn7-Cm-invCFLAG(LVA), pGPTn7-Cm-invCFLAG(AAV), or pGPTn7-Cm-invCFLAG(ASV). These constructs were used to insert *invC-FLAG*, *invC-*FLAG(LVA), *invC-*FLAG(AAV), or *invC-*FLAG(ASV) at the *att*Tn*7* site in the *S*. Typhimurium SL1344 Δ*invC* chromosome by conjugation (35). Lastly, the Cm^r^ cassette, which is flanked by flippase recognition target (FRT) sites, was excised from the chromosome by using pCP20 (68).

SL1344 Δ*hilE*::FRT was created by P22 transduction from *S*. Typhimurium 14028 Δ*hilE*::kan (70), followed by excision of the Kan^r^ cassette using pCP20 (68). The Δ*hilE*::FRT *zjg8112*::*tetRA-hilE* strain was constructed as follows: the *tetRA* cassette was amplified from Tn*10*d-Tet by using primers JRG-092 and JRG-093 and recombined into *S*. Typhimurium strain 14028 upstream of *hilE* via λ Red-mediated recombination to create a *tetA-hilE*^+^ operon under the control of the *tetA* promoter. The *tetRA-hilE*^*+*^ construct was amplified from this strain by using primers JRG-094 and JRG-095 and subsequently recombined via λ*-*Red into *S*. Typhimurium 14028 between *purA* and *yjeB*-*nsrR*. The allele number for this insertion is *zjg8112*. Finally, *tetRA-hilE*^+^ was moved by P22 transduction into SL1344 Δ*hilE*::FRT.

The pACYC177-SopB-CyaA-SigE plasmid has been described previously (71). pBAD30-*sipB* and pBAD30-*sipC* (47) are derivatives of pBAD30 (Amp^r^) (33) and contain the native *sipB* and *sipC* RBS, respectively. For fluorescence detection of intracellular bacteria, *S*. Typhimurium strains were either (i) electroporated with pFPV-mCherry (37) (which constitutively expresses mCherry under the control of the *S*. Typhimurium *rpsM* promoter) or (ii) transduced with P22 lysate derived from *S*. Typhimurium SL1344 *glmS*::*Ptrc*-*mCherryST* (56) (which constitutively expresses mCherry that is codon optimized for *S*. Typhimurium under the control of the *trc* promoter), followed by removal of the Cm^r^ cassette by using pCP20 (68).

### Tissue culture.

HeLa (ATCC CCL-2), HCT 116 (ATCC CCL-247), and C2BBe1 (ATCC CRL-2102) epithelial cells and J774A.1 (ATCC TIB-67) macrophage-like cells were purchased from the American Type Culture Collection (ATCC) and maintained in complete growth medium containing 10% (vol/vol) heat-inactivated fetal calf serum (Invitrogen), as recommended by ATCC. Cells were used within 15 passages of receipt from ATCC.

### Induction conditions and epithelial cell infections.

*S*. Typhimurium strains were grown under T3SS1-inducing conditions as previously described (14) (3.5-h subcultures in LB-Miller broth, with shaking at 220 rpm), and the invasion efficiency of bacterial strains was determined in gentamicin protection assays, following an established protocol (32). Invasion efficiency (the percentage of the inoculum internalized) for each strain is expressed as a percentage of the invasion efficiency for wild-type bacteria (set to 100%). For gene induction in broth, *S*. Typhimurium subcultures were treated with (i) 0.2% (wt/vol) arabinose (Sigma) for 1 h (2.5 h to 3.5 h of subculture growth) or (ii) 100 ng/ml ATc (Acros Organics) for 1.5 h (2 to 3.5 h of subculture growth) immediately prior to infection. HeLa cell infections were performed as previously described (32). For gene induction inside mammalian cells, (i) 1% (wt/vol) arabinose was added at *t0* and maintained throughout infection, or (ii) 300 ng/ml ATc was added after the 10-min internalization step and maintained throughout infection.

### Determination of variant InvC half-lives.

For determination of the InvC half-life in liquid broth, 100 ng/ml ATc was added for 1 h (at 1 to 2 h of subculture growth). ATc was then washed out by repeated centrifugation and washing of bacterial cultures in phosphate-buffered saline (PBS). Chloramphenicol (30 µg/ml) was added to stop *de novo* protein synthesis (*t*0), and cultures were then incubated statically at 37°C. At the indicated times, samples were collected and centrifuged, and the bacterial pellet was resuspended in hot 1.5× SDS-PAGE sample buffer. Samples were transferred to a heat block set to 95°C for 5 min. For determination of the InvC half-life in mammalian cells, HeLa cells were seeded in 6-well plates at 2.2 × 10^5^ cells/well at 24 h prior to infection. Where appropriate, bacterial subcultures were induced with ATc as described above. The MOI was adjusted for each strain so that internalized CFU were equivalent to counts for wild-type *S*. Typhimurium (determined in a gentamicin protection assay at 1 h p.i.). Chloramphenicol (60 µg/ml) was added to infected cells at 30 min p.i., and at the indicated times monolayers were washed twice in PBS and solubilized in 150 µl hot 1.5× SDS-PAGE sample buffer.

### Immunoblotting.

Proteins were separated by 10% SDS-PAGE and transferred to 0.45-µm nitrocellulose membranes. Membranes were blocked at room temperature for 1 h in Tris-buffered saline with 5% milk powder containing 0.1% (vol/vol) Tween 20 (TBST). Blots were then incubated with the following primary antibodies overnight at 4°C: mouse monoclonal anti-FLAG M2, affinity isolated (1:2,000 dilution; Sigma), mouse monoclonal anti-SipA 1A9 (1:500), anti-SipB 1A10 (1:5,000), anti-SipC 1A5 (1:1,000) (all three of these antibodies were kindly provided by Ciaran Finn and Olivia Steele-Mortimer, Rocky Mountain Laboratories), mouse polyclonal anti-SipD (1:2,000 dilution; kindly provided by Francisco Martinez-Becerra, University of Kansas), and mouse monoclonal anti-*E. coli* DnaK clone 8E2/2 (1:20,000 dilution; Enzo). Horseradish peroxidase-conjugated secondary antibodies (Cell Signaling) were used at a 1:10,000 dilution, and blots were developed using a West Femto detection kit (Pierce). Bio-Rad Image Lab software was used to quantify protein bands by densitometry.

### Determination of intracellular T3SS1 activity.

HeLa cells were seeded in 6-well plates at 2.4 × 10^5^ cells/well 24 h prior to infection. Bacterial subcultures were induced with ATc or arabinose where appropriate. The MOI was adjusted for each strain so that the internalized CFU was equivalent to that of wild-type *S*. Typhimurium (determined in the gentamicin protection assay at 1 h p.i.). At 1 h and 8 h p.i., monolayers were washed with PBS and resuspended directly in 100 µl boiling 1.5× SDS-PAGE sample buffer.

Alternatively, HeLa cells were seeded at 5 × 10^4^ cells/well in 24-well plates 24h prior to infection. Bacterial subcultures of strains bearing pACYC177-SopB-CyaA-SigE were induced with ATc where appropriate. The MOI was adjusted for each strain so that internalized CFU were equivalent to that of wild-type *S*. Typhimurium (determined in the gentamicin protection assay at 1 h p.i.). Monolayers were washed with PBS at 30 min and 8 h p.i., then lysed and processed for cAMP quantification as described previously (32). cAMP was measured using the Amersham cAMP Biotrack enzyme immunoassay system (GE Healthcare BioScience) according to the manufacturer’s instructions for the nonacetylation procedure.

### Fluorescence microscopy.

HeLa cells were seeded on acid-washed glass coverslips at 6 × 10^4^ cells/well in 24-well plates 24 h prior to infection. *S*. Typhimurium strains constitutively expressing chromosomal (*glmS*::*Ptrc-mCherryST*) or plasmid-borne mCherry (pFPV-mCherry) were subcultured under T3SS1-inducing conditions and induced with ATc or arabinose, as described above, where required. The MOIs were adjusted for each strain so that the number of internalized bacteria was equivalent to the number of wild-type *S*. Typhimurium (determined at 1 h p.i.). At each indicated time point, monolayers were washed once with PBS and then fixed in 2.5% (wt/vol) paraformaldehyde for 10 min at 37°C. Cells were incubated with Alexa Fluor 488-phalloidin (1:200 dilution; Thermo Fisher Scientific) diluted in 10% (vol/vol) normal goat serum (Gibco)–0.2% (wt/vol) saponin (Calbiochem)–PBS for 15 min at room temperature. Coverslips were mounted in Mowiol on glass slides and viewed on a Leica DM4000 upright fluorescence microscope. The number of bacteria in each cell was blindly scored for ≥60 infected cells/experiment. Immunostaining for SipC was performed as we recently described (32).

### Gentamicin protection, CHQ resistance, and bacterial egress assays.

The gentamicin protection, CHQ resistance, and bacterial egress assays were performed as described previously (44). CHQ concentrations used were as follows; HeLa, 400 µM; HCT 116, 400 µM; J774A.1, 400 µM; C2BBe1, 1 mM and 600 µM at 90 min p.i. and 7 h p.i., respectively.

### Statistical analysis.

All experiments were conducted on at least three separate occasions, and results are presented as means ± SD unless otherwise stated. Statistical analyses were performed using analysis of variance (ANOVA) with Dunnett’s *post hoc* test (KaleidaGraph). *P* values of ≤0.05 were considered significant.
