# Supplementary material for: Controlled Activity of the Salmonella Invasion-Associated Injectisome Reveals Its Intracellular Role in the Cytosolic Population
Source: mBio. 2017 Dec 5;8(6):e01931-17. doi: 10.1128/mBio.01931-17 (PMC5717391; doi:10.1128/mBio.01931-17)
Supplement: FIG S1 [file mbo006173612sf1.pdf]

A

Cell line	Invasion efficiency of Δ*prgI* (% WT)
HCT116	0.11±0.04
HeLa	0.64±0.4
C2BBe1	14.1±2.0
J774A.1	30.4±12.1

B

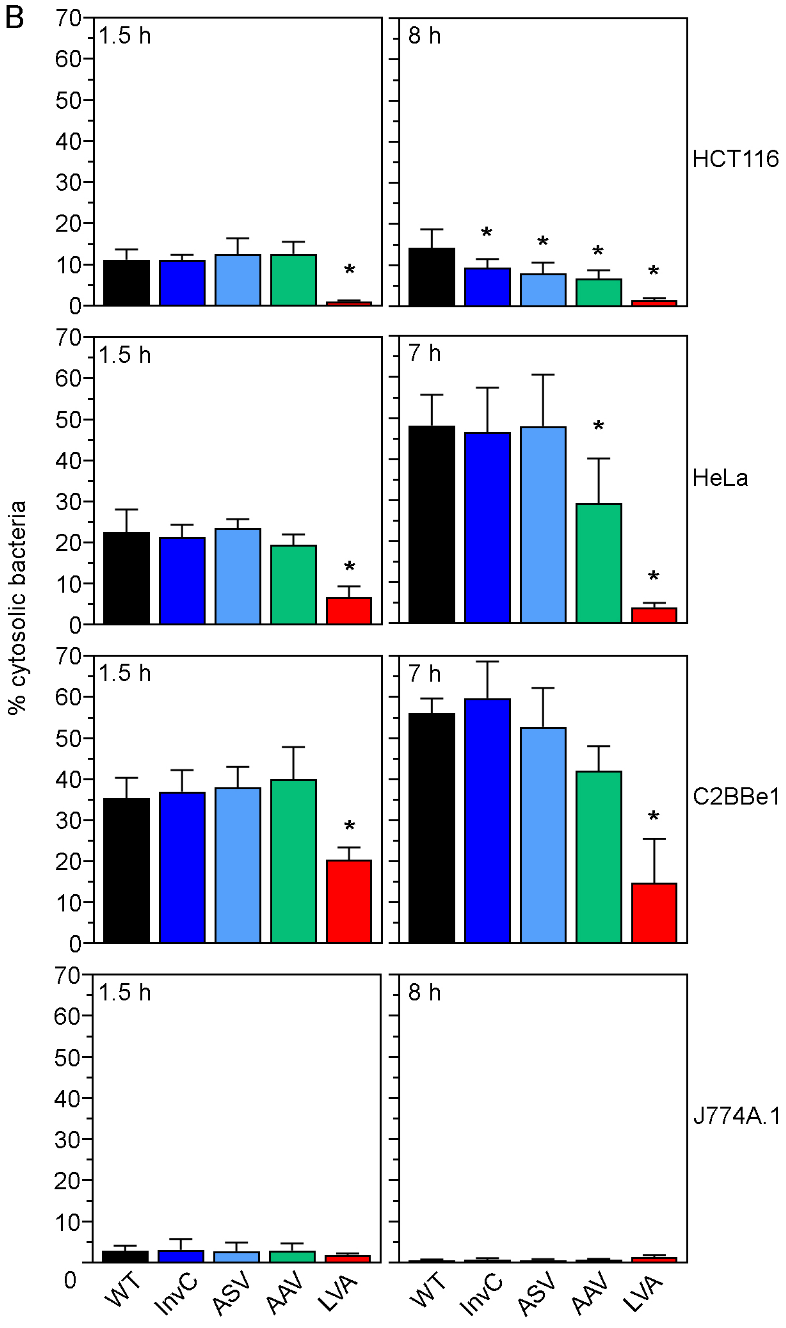

FIG S1: Effect of InvC destabilization in cell lines with differential requirement for T3SS1-mediated entry (A) Invasion efficiency of a T3SS1 mutant (Δ*prgI*) as determined by gentamicin protection assay in indicated cell lines at 1 h p.i., expressed as a percentage of the invasion efficiency for wild type bacteria in each cell line (set to 100%). HCT116, human colonic epithelial cells; HeLa, human cervical epithelial cells; Caco-2 C2BBe1, human colonic epithelial cells; J774A.1, mouse macrophage-like cells. (B) CHQ resistance assays in various cell lines. Bacterial subcultures were grown in the presence of 100 ng/ml ATc for 1.5 h prior to infection to induce synthesis of InvC-FLAG. MOIs were adjusted so that equivalent numbers of bacteria were internalized, and the proportion of cytosolic bacteria was determined at the indicated time points by CHQ resistance assay (mean ± SD, n≥3 independent experiments). Asterisks denote data significantly different from WT bacteria.
