# Supplementary material for: Controlled Activity of the Salmonella Invasion-Associated Injectisome Reveals Its Intracellular Role in the Cytosolic Population
Source: mBio. 2017 Dec 5;8(6):e01931-17. doi: 10.1128/mBio.01931-17 (PMC5717391; doi:10.1128/mBio.01931-17)
Supplement: FIG S2 [file mbo006173612sf2.pdf]

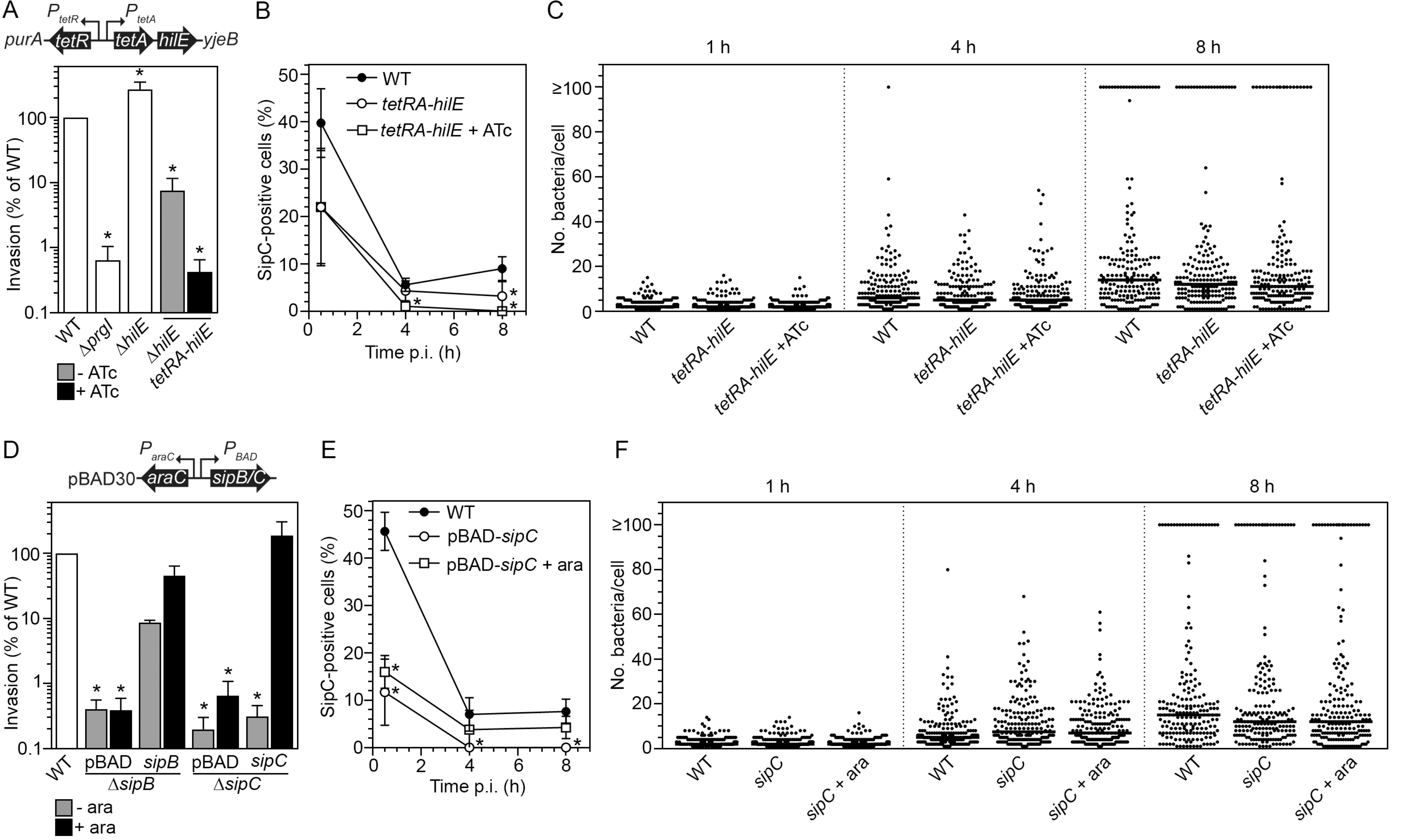

FIG S2: Controlled expression of *hilE* or *sipC* does not affect intracellular replication. (A) Cartoon depicts regulated *hilE* expression. *hilE* is under the control of P_{tetA} and inserted in the chromosome of a Δ*hilE* mutant between *purA* (SL1344_4299) and *yjeB/nsrR* (SL1344_4300). Invasion efficiency of *S.* Typhimurium wild type (WT), Δ*prgI* (a T3SS1 null mutant), Δ*hilE*, and Δ*hilE tetRA::hilE*. Where indicated, subcultures were grown in the absence (grey) or presence (black) of 100 ng/ml ATc. Invasion efficiency (percentage of inoculum internalized) was determined by gentamicin protection assay in HeLa cells at 1 h p.i. and expressed as a percentage of the invasion efficiency for wild type bacteria (set to 100%). (B) Time course of SipC delivery into epithelial cells. HeLa cells were infected with *S.* Typhimurium WT and Δ*hilE tetRA-hilE* (ATc-induced) bacteria (chromosomal P_{trc}-*mCherry* strains). Where indicated (open squares), 300 ng/ml ATc was added to infected cells at 10 min p.i. and maintained throughout infection. Monolayers were fixed at 0.5 h, 4 h and 8 h p.i. and immunostained for SipC. The percentage of infected cells positive for SipC signal was scored by fluorescence microscopy. (C) Single-cell analysis of intracellular proliferation in epithelial cells. HeLa cells were infected as in (B), fixed at 1 h, 4 h and 8 h p.i. and the number of bacteria in each infected cell was scored by fluorescence microscopy. Cells with ≥100 bacteria contain cytosolic S. Typhimurium. Each dot represents one infected cell and data from 3 independent experiments is shown (>180 cells in total). (D) Cartoon depicting regulated *sipB* and *sipC* expression. These genes are under the control of the *araBAD* promoter (P_{BAD}) in the pBAD30 vector. Invasion efficiency of S. Typhimurium wild type (WT) compared to Δ*sipB* pBAD30, Δ*sipB* pBAD30-*sipB*, Δ*sipC* pBAD30 and Δ*sipC* pBAD30-*sipC*. Where indicated, subcultures were grown in the absence (grey) or presence (black) of 0.2% (w/v) arabinose. (E) Time course of SipC delivery into epithelial cells. Cells were infected with WT and Δ*sipC* pBAD30-*sipC* (arabinose-induced) bacteria (chromosomal P_{trc}-*mCherry* strains). Arabinose (1% (w/v)) was added at t_0 where indicated (open squares) and maintained throughout infection. (F) Single-cell analysis of intracellular proliferation in epithelial cells. HeLa cells were infected as in (E). Cells with ≥100 bacteria contain cytosolic *S.* Typhimurium. Each dot represents one infected cell and data from 3 independent experiments is shown (>180 cells in total). For all panels, asterisks indicate data significantly different from WT
