# Supplementary material for: Controlled Activity of the Salmonella Invasion-Associated Injectisome Reveals Its Intracellular Role in the Cytosolic Population
Source: mBio. 2017 Dec 5;8(6):e01931-17. doi: 10.1128/mBio.01931-17 (PMC5717391; doi:10.1128/mBio.01931-17)
Supplement: FIG S3 [file mbo006173612sf3.pdf]

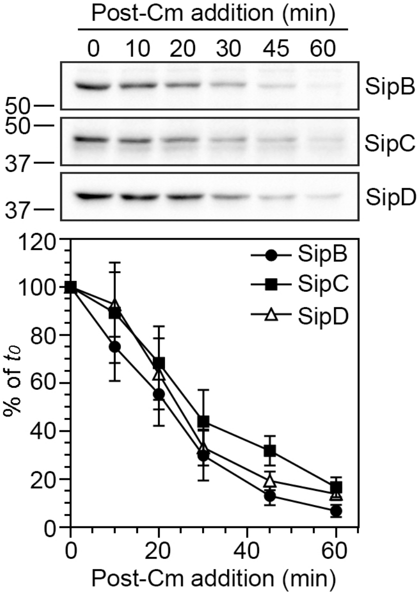

FIG S3: SipB, SipC and SipD have short half-lives in mammalian cells. HeLa epithelial cells were infected with S. Typhimurium SL1344 sipB::3xFLAG, sipC::3xFLAG or sipD::3xFLAG strains and at 30 min p.i. chloramphenicol (60 µg/ml) was added to stop de novo protein synthesis (t_0). Lysates were collected at the indicated times and subject to immunblotting with α-FLAG antibodies. Graph depicts densitometric analysis from 3 independent experiments.
