# Supplementary material for: Controlled Activity of the Salmonella Invasion-Associated Injectisome Reveals Its Intracellular Role in the Cytosolic Population
Source: mBio. 2017 Dec 5;8(6):e01931-17. doi: 10.1128/mBio.01931-17 (PMC5717391; doi:10.1128/mBio.01931-17)
Supplement: TABLE S1 [file mbo006173612st1.docx]

**Table S1: Oligonucleotides used for cloning**

| **Name** | **Sequence (5’ to 3”)** | **Description** |
| --- | --- | --- |
| Kpn-invC-F | GGGGTACCGTCAGACCTCTGGCAGTA | SL1344 *∆invC* |
| Xma-invC-R2 | CCCCCCGGGGCGTTATCGGTTACTTCACTAACG | SL1344 *∆invC* |
| dinvC-OLF | **ATG**AAAACACCTCGTGTAGCGCAGTATTCATCC | SL1344 *∆invC* |
| dinvC-OLR | TGAATACTGCGCTACACGAGGTGTTTT**CAT**CTC | SL1344 *∆invC* |
| Kpn-dsopA-F | GGGGTACCCATGTCAACACGATAAAACCT | SL1344 *∆sopA* |
| Xma-dsopA-R | CCCCCCGGGCTCCGGCTCGCCAATAAC | SL1344 *∆sopA* |
| dsopA-OLF | TCAGGCGCAATTAATTTTTCATCCATCCTGCCACTGGCC | SL1344 *∆sopA* |
| dsopA-OLR | CAGTGGCAGGATGGATGAAAAATTAATTGCGCCTGATGA | SL1344 *∆sopA* |
| sipA-KO-F | GATAACAGAAGAGGATATTAATA**ATG**GTTACAAGTGTAAGGTGTAGGCTGGAGCTGCTTCG | SL1344 ∆*sipA*::kan |
| sipA-KO-R | CATCTTTCCCGGTTAA**TTA**ACGCTGCATGTGCAAGCCATCAACGCATATGAATATCCTCCTTAG | SL1344 ∆*sipA*::kan |
| sopD-KO-F | TATTGAATAATATAAATTTGAAGGAAAATATT**ATG**CCAGTCACTTGTAGGCTGGAGCTGCTTCG | SL1344 ∆*sopD*::kan |
| sopD-KO-R | TTTTAAATTGGTTATATTACTGACTATCT**TTA**TGTCAGTAATACATATGAATATCCTCCTTAG | SL1344 ∆*sopD*::kan |
| Kpn-tetR-R | GGGGTACC**TTA**AGACCCACTTTCACATTTAAG | pGPTn7-Cm-invC  pGPTn7-Cm-invCFLAG  pGPTn7-Cm-invCFLAG(LVA) pGPTn7-Cm-invCFLAG(AAV) pGPTn7-Cm-invCFLAG(ASV) |
| tetARBS-invC-R | TAAACGAGGTGTTTT**CAT**TTCACTTTTCTCTATCACT | pGPTn7-Cm-invC |
| tetARBS-invC-F | GTGATAGAGAAAAGTGAA**ATG**AAAACACCTCGTTTACTG | pGPTn7-Cm-invC |
| XhoI-invC-R | CCGCTCGAG**TTA**ATTCTGGTCAGCGAATGCATTC | pGPTn7-Cm-invC |
| XhoI-invCFLAG-R | CCGCTCGAG**TTA**TTTATCGTCGTCATCTTTGTAGTCGATATCATGATCTTTATAATCACCGTCATGGTCTTTGTAGTCATTCTGGTCAGCGAATGCATTC | pGPTn7-Cm-invCFLAG |
| XhoI-FLAGLVA-R | CCGCTCGAG**TTA**AGCTACTAAAGCGTAGTTTTCGTCGTTTGCTGCAGGCCTTTTATCGTCGTCATCTTTGTAGTC | pGPTn7-Cm-invCFLAG(LVA) |
| XhoI-FLAGAAV-R | CCGCTCGAG**TTA**AACTGCTGCAGCGTAGTTTTCGTCGTTTGCTGCAGGCCTTTTATCGTCGTCATCTTTGTAGTC | pGPTn7-Cm-invCFLAG(AAV) |
| XhoI-FLAGASV-R | CCGCTCGAG**TTA**AACGCTTGCAGCGTAGTTTTCGTCGTTTGCTGCAGGCCTTTTATCGTCGTCATCTTTGTAGTC | pGPTn7-Cm-invCFLAG(ASV) |
| JRG-092 | GTTCTTTCATTGTTGGCGTTCCGTCCTTGTTTAAGACCCACTTTC | *tetRA-hilE^+^* |
| JRG-093 | TCCGTGATTATCATGCGGCAAAGCAGGCTGCTAAGCACTTGTCTC | *tetRA-hilE^+^* |
| JRG-094 | GCTTGCCGGGCTAACGCGATTATTTTCGCTTTAAGACCCACTTTC | SL1344 ∆*hilE*::FRT *zjg8112*::*tetRA-hilE* |
| JRG-095 | GTAATGGCTTACCCGCCATCCTGATAGAGCTTGTTTTGTCCTCATGCG | SL1344 ∆*hilE*::FRT *zjg8112*::*tetRA-hilE* |
| XhoI-SipAcomp-F | CCGCTCGAGTATGAAACGAATCAGCGCGCA | pGPTn7-Cm-*sipA* |
| SipACyaA-OLR | AGTCCTTACACTTGTAAC**CAT**TACTTACTCCTGTTATCTGTC | pGPTn7-Cm-*sipA* |
| SipACyaA-OLF | AGATAACAGGAGTAAGTA**ATG**GTTACAAGTGTAAGGACTCAG | pGPTn7-Cm-*sipA* |
| SmaI-SipAcomp-R | TCCCCCGGGTAA**TTA**ACGCTGCATGTGCAA | pGPTn7-Cm-*sipA* |
| Xho-SopBcomp-F | CCGCTCGAGCGCGAATTCTATCTGTTCAAG | pGPTn7-Cm-*sopBsigE* |
| Sma-SigEcomp-R | TCCCCCGGGAAA**TTA**TGCATAATGCTCTTTCAA | pGPTn7-Cm-*sopBsigE* |

Engineered restriction sites are underlined. Start and stop codons are in **bold**.
